# Overview of Early ChatGPT’s Presence in Medical Literature: Insights From a Hybrid Literature Review by ChatGPT and Human Experts

**DOI:** 10.7759/cureus.37281

**Published:** 2023-04-08

**Authors:** Omar Temsah, Samina A Khan, Yazan Chaiah, Abdulrahman Senjab, Khalid Alhasan, Amr Jamal, Fadi Aljamaan, Khalid H Malki, Rabih Halwani, Jaffar A Al-Tawfiq, Mohamad-Hani Temsah, Ayman Al-Eyadhy

**Affiliations:** 1 Collage of Medicine, Alfaisal University, Riyadh, SAU; 2 Computer Sciences, Universiti Sains Malaysia, Penang, MYS; 3 College of Medicine, Alfaisal University, Riyadh, SAU; 4 Pediatric Nephrology, King Saud University, Riyadh, SAU; 5 Family and Community Medicine, King Saud University, Riyadh, SAU; 6 Critical Care, King Saud University, Riyadh, SAU; 7 Otolaryngology, King Saud University, Riyadh, SAU; 8 Clinical Sciences, University of Sharjah, Sharjah, ARE; 9 Specialty Internal Medicine and Quality, Johns Hopkins Aramco Healthcare, Dhahran, SAU; 10 Pediatric Intensive Care, King Saud University, Riyadh, SAU

**Keywords:** machine learning (ml), hybrid method, medical practice, artificial intelligence in medicine, ethical considerations, diagnostic decision-making, chatgpt, medical writing, generative language model, medical education

## Abstract

ChatGPT, an artificial intelligence chatbot, has rapidly gained prominence in various domains, including medical education and healthcare literature. This hybrid narrative review, conducted collaboratively by human authors and ChatGPT, aims to summarize and synthesize the current knowledge of ChatGPT in the indexed medical literature during its initial four months. A search strategy was employed in PubMed and EuropePMC databases, yielding 65 and 110 papers, respectively. These papers focused on ChatGPT's impact on medical education, scientific research, medical writing, ethical considerations, diagnostic decision-making, automation potential, and criticisms. The findings indicate a growing body of literature on ChatGPT's applications and implications in healthcare, highlighting the need for further research to assess its effectiveness and ethical concerns.

## Introduction and background

ChatGPT, a new artificial intelligence platform created by OpenAI, has undoubtedly taken the world by storm in no time. Released in November 2022, this artificial intelligence chatbot uses a neural network machine learning model and generative pre-trained transformer (GTP) to pull from a significant amount of data to formulate a conversation-style response in various written content, for a multitude of domains, from history to philosophy, science to technology, banking, marketing, entertainment, in the form of articles, social media posts, essays, computer programming codes and emails [1, 2]. ChatGPT has taken its role in academia, particularly in medical education. A recent study shows that ChatGPT has reached the standard of passing a third-year medical student exam [3]. ChatGPT is a state-of-the-art AI model that can generate human-like text in response to user queries [4]. Thus, shortly, a ChatGPT-supported application is a potential source for an interactive tool in medical education to support learning [3, 5]. Exploring what the new ChatGPT publications address has the potential to enlighten healthcare providers and medical education specialists on ChatGPT's contributions to healthcare and medical education. This review aims to summarize and synthesize the current knowledge of ChatGPT in healthcare and medical education during the last four months since its launch.

## Review

Methodology

This paper presents a hybrid narrative review on the topic of ChatGPT in medical education and medical literature, aiming to identify gaps in the existing literature and provide a comprehensive overview of the current state of research [6]. Our hybrid approach combines the conventional narrative review methodology with the assistance of ChatGPT in analyzing and synthesizing the retrieved abstracts. PubMed and EuropePMC were searched using the keyword "ChatGPT".

Inclusion Criteria

The inclusion criteria included articles discussing ChatGPT in the context of medical education, medical literature, or medical practice, including its applications, potential benefits, and limitations; articles published in peer-reviewed journals or conference proceedings, ensuring a certain level of quality and credibility; studies employing quantitative, qualitative, or mixed-methods approaches to assess the impact or implications of ChatGPT in the medical domain; commentaries, editorials, or other non-research articles that provided early insights into ChatGPT in medical education or medical literature; and articles published in English between November 01, 2022, and February 27, 2023.

Exclusion Criteria

The exclusion criteria included articles focusing on ChatGPT applications in non-medical domains or unrelated contexts; articles published in languages other than English, as our review team was not equipped to assess non-English sources accurately; articles published outside the specified date range; and articles published in other databases.

Upon retrieval of relevant titles and abstracts, we input them into ChatGPT, prompting it to discern and emphasize the principal themes among the selected papers. The human authors then meticulously analyzed ChatGPT's output, cross-referencing it with the original articles to ensure accuracy and comprehensiveness. This hybrid approach allowed us to leverage the capabilities of ChatGPT in the review process while maintaining human oversight for quality and interpretation. The following summary of the review's findings was then compiled, providing insights into the current state of ChatGPT's application in medical education and literature. 

Results

**Figure 1:**
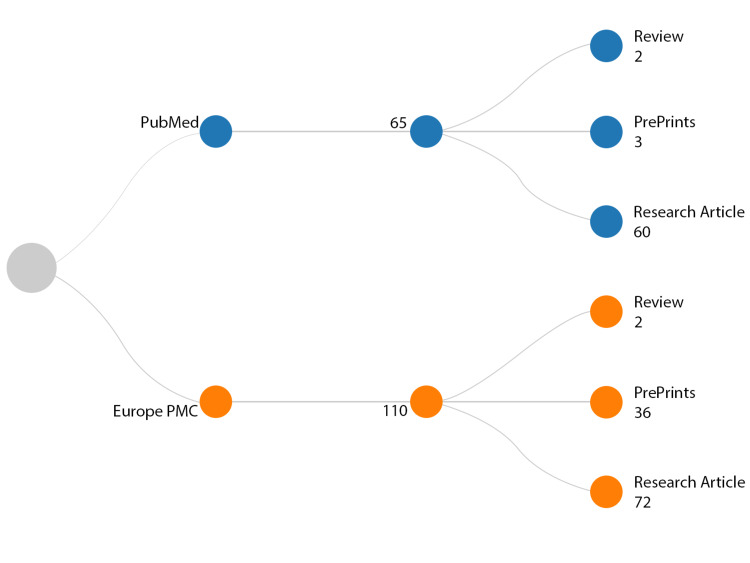
Overview of retrieved studies

The PubMed search uncovered 65 papers, comprising 60 research articles, three preprints, and two reviews. Interestingly, one of the papers listed ChatGPT as a co-author [7]. Of the total papers, five were listed in PubMed in 2022, with 60 published since January 2023. On Europe PMC, 110 papers were found, consisting of 72 research articles, 36 preprints, two reviews, and five papers listing ChatGPT as a co-author. Out of these, 11 papers were published in 2022 and 99 since January 2023 (Figure 1).

Among the 65 papers selected on PubMed since December 2022, we found a focus on the utilization of ChatGPT in various medical fields, including medical education, scientific writing, research, and diagnostic decision-making. These papers can be categorized into eight themes based on their abstracts: (1) medical writing, (2) medical education, (3) diagnostic decision-making, (4) public health, (5) scientific research, (6) ethical considerations of ChatGPT use, (7) ChatGPT's potential to automate medical tasks, and (8) criticism of ChatGPT's usage (summarized in Table 1). These themes are supported by the papers' topics, including ChatGPT's ability to provide medical information, its impact on medical education and research, its potential to replace human-written papers, its ethical implications, and its limitations in providing accurate diagnoses.

**Table 1:** Summary of key findings from the review

Theme of ChatGPT	Key findings
ChatGPT's impact on medical education	Enhances learning, interpretation, and recall of medical information. Provides reliable information in specific medical fields. Concerns about undermining clinical reasoning.
Use in scientific research	Generates coherent and readable content. Raises ethical concerns regarding authorship, transparency, and accountability. Concerns about accuracy and promoting conspiracy theories.
Use in medical writing	Popular tool for generating medical content. Challenges traditional roles of authorship. Concerns about accuracy and ethical implications.
Ethical considerations	Raises concerns about authorship, accountability, and transparency. Potential for generating misleading or inaccurate information and promoting harmful beliefs. Importance of human oversight and transparency in its use.
Role in diagnostic decision-making	Improves efficiency and reduces errors in decision-making. Concerns about accuracy, perpetuating biases, and need for human oversight.
Role in public health	Provides reliable estimates in public health research. Concerns about potential misleading information and amplification of harmful beliefs.
Potential for automating medical tasks	Concerns about undermining the value of human expertise. Need for human oversight.
Criticisms of use	Concerns about accuracy and potential to promote conspiracy theories. Challenges traditional roles of authorship and transparency. Ethical concerns and potential to promote bias in decision-making.

Implications of ChatGPT in Medical Writing

ChatGPT is becoming a popular tool for generating medical writing, leading to discussions on its reliability and ethical considerations. It is argued that ChatGPT may shape the future of medical writing, as it can generate papers with high coherence and readability [8]. However, there are concerns about its accuracy as it may produce inaccurate information or promote conspiracy theories. Additionally, the use of ChatGPT raises ethical concerns as it challenges the traditional role of authorship, transparency, and accountability in scientific research [9, 10].

ChatGPT and Medical Education

ChatGPT has been explored as a tool for medical education, particularly in enhancing learning, interpretation, and recall of medical information [11, 12]. Some studies have shown that ChatGPT can provide reliable information comparable to medical students in specific medical fields such as parasitology. However, it is crucial to ensure the standardization, validity, and reliability of the information fed to ChatGPT to maintain its effectiveness, particularly considering that its current data is up to 2021.

Also, medical students' dependence on ChatGPT can undermine clinical reasoning despite its potential benefits [13]. Moreover, ChatGPT's responses may lack context or may not be tailored to the specific needs of individual learners, which is a limitation considering the complexity of the medical education process and the diverse learning needs and styles of students.

ChatGPT and Diagnostic Decision-making

ChatGPT has been used as an adjunct in diagnostic decision-making in radiology, cardiology, and urology, where it improved efficiency and reduced errors in decision-making. However, there are concerns about the accuracy of the generated output, the potential for ChatGPT to perpetuate biases in diagnoses, and the need for human oversight in its use [14-17]. As an AI language model, ChatGPT can only provide information based on the data it has been trained on, so its responses should be regarded as a source of information rather than a substitute for professional medical advice.

ChatGPT and Public Health

ChatGPT has been used in public health research to analyze population-level data on vaccine effectiveness, COVID-19 conspiracy theories, and compulsory vaccination. Some studies have shown that ChatGPT can provide reliable estimates in public health research, provided that the input data is valid and accurate. However, concerns persist about the potential for misleading information and the amplification of harmful beliefs if the input data is flawed or biased [18].

Implications of ChatGPT in Science and Research

ChatGPT has been explored as a tool for generating scholarly content, including scientific research and academic publishing, such as drafting articles, abstracts, and research proposals [19]. Some argue that ChatGPT can improve the efficiency and speed of scientific writing by streamlining the writing process and assisting with literature reviews, data interpretation, and hypothesis generation. However, there are concerns about its accuracy, the potential to undermine the value of human expertise, and challenges to traditional authorship and transparency in scientific research [20]. Discussions on the implications of ChatGPT in science and research include the need for ethical considerations, the importance of human oversight, and the potential to promote transparency and efficiency in scientific writing [21].

Ethical Considerations of ChatGPT

ChatGPT has raised ethical concerns regarding authorship, accountability, and transparency in scientific research and medical writing [9]. In the context of medical education, there are additional concerns about the potential for bias or misinformation, as AI models can amplify existing biases present in the training data, and there is a risk that ChatGPT's responses may perpetuate these biases. Therefore, it is crucial to ensure that ChatGPT is trained on unbiased and diverse data and that its responses are carefully monitored for accuracy and fairness. Furthermore, discussions on the ethical considerations of ChatGPT include the need for transparency in its use, the importance of human oversight, and the potential to promote bias in decision-making [8, 10].

ChatGPT's Potential to Automate Medical Tasks

ChatGPT, alongside other large language models, has raised discussions on the future of programming and its potential to replace programmers. ChatGPT has been used in generating code, and some studies have shown that it can improve the efficiency and speed of programming. However, there are concerns about the potential to undermine the value of human expertise in programming, the need for human oversight in its use, and the risk of the tool being hacked, compromising genuine data [20].

Criticism of ChatGPT Usage

Incorporating AI and large language models (LLMs) such as ChatGPT into healthcare necessitates meticulous attention to emerging challenges and concerns. While ChatGPT's adeptness in mimicking human dialogue presents opportunities for improving patient-provider interactions and potentially enhancing patient adherence to prescribed treatments, the model's limited grasp of context and nuance, along with its failure to consistently recognize its own limitations, highlights the perils of unsupervised deployment in clinical settings [22, 23].

Research examining ChatGPT's application in medical scholarship shows that the language model exhibits a disconcerting tendency to produce plausible yet erroneous content (i.e., hallucinations), including spurious references, thereby jeopardizing scientific integrity and the dissemination of accurate information. [24]. Furthermore, despite the potential advantages of utilizing ChatGPT for peer-to-peer mental health support, its vulnerability to bias and perpetuating stereotypes demands rigorous ethical and legal scrutiny, particularly when AI-assisted clinicians commit errors, or patients eschew professional consultation in favor of AI-generated medical counsel [25]. The urgent need for comprehensive training based on expert annotations, formal evaluations, and stringent regulatory safeguards to ensure alignment with clinical performance benchmarks and avert detrimental outcomes is paramount. These considerations emphasize the necessity for interdisciplinary collaboration and judicious incorporation of AI and LLMs into healthcare systems, prioritizing the augmentation of human expertise to pursue optimal patient outcomes [26].

Discussion

The positive aspects of the medical journal's publications on ChatGPT include the in-depth analysis of ChatGPT and its capabilities and the potential benefits of using chatbots in various industries [27]. The publications also provide a comprehensive list of references, which can be useful for further research. ChatGPT represents a significant leap forward in natural language processing (NLP), employing a large-scale, pre-trained neural network to generate human-like responses to user queries [28].

Furthermore, its ability to personalize responses to a broad spectrum of medical queries makes it an essential tool in the medical field. This is possible due to the accuracy and flexibility of ChatGPT achieved through transfer learning, a deep learning technique that fine-tunes responses for specific medical domains [29]. This approach allows ChatGPT to analyze the context in which a query is used and generate tailored responses for the specific user [30]. However, it is important to note that ChatGPT's responses are limited to the data it has been trained on, and its knowledge is based on patterns it has learned from large amounts of text data. Therefore, its responses may not always be up-to-date or accurate for every scenario.

Additionally, since ChatGPT is not a live system and its responses are generated based on the data it has learned from, it may not always have access to the latest information or developments in a given field, especially in rapidly evolving areas such as medicine or healthcare. Despite these limitations, one of the notable advantages of ChatGPT over previous NLP tools and other chatbots is the generation of human-like responses. Traditional chatbots rely on a linear, decision-tree approach, which provides only predefined answers to a limited set of questions.

In contrast, ChatGPT can respond to more extensive queries with personalized and natural-sounding responses, allowing for enhanced human-like interaction. [31-33]. This is particularly critical in the medical field, where there is a need for clear and accurate communication of medical information to patients and healthcare professionals [34].

Furthermore, ChatGPT's ability to provide a higher range of interactions is another advantage over previous NLP tools and chatbots. Traditional chatbots are limited to predefined responses, whereas ChatGPT can provide more diverse responses to user queries. This is possible due to the neural network's size and complexity, allowing ChatGPT to process and analyze vast data [35]. As a result, ChatGPT can provide more informative and detailed responses, which can be especially useful in the medical field [36]. However, some negative aspects of the publication include the limited scope of the analysis and the lack of empirical evidence to support the claims [37]. Despite its numerous advantages, ChatGPT has some limitations and disadvantages compared to other available AI and chatbots in the medical field. One significant drawback is its high computational cost [33, 38]. ChatGPT employs a large-scale neural network, which requires substantial computational power and considerable memory, making it more challenging to deploy and integrate into smaller medical applications.

Moreover, the model's size can make fine-tuning for a specific domain difficult, leading to inconsistencies in the generated responses [39]. Another limitation of ChatGPT is its inability to incorporate external knowledge sources, limiting its accuracy in the medical field. In contrast to some other AI and chatbots, ChatGPT is not designed to extract information from external sources such as medical journals or textbooks, which can provide additional context and background knowledge [22]. Furthermore, ChatGPT may not generate responses incorporating the latest medical research or best practices without its ability to integrate updated and reliable data, considering that its original data training was up until 2021 [40]. Lastly, ChatGPT's responses can be inconsistent, leading to confusion and mistrust in the medical field [41]. As it is trained on a vast corpus of text, it can generate contradictory or inconsistent responses with medical guidelines. This can be especially problematic in the medical field, where accuracy and consistency are critical. Although ChatGPT can be fine-tuned to improve its accuracy, this process can be time-consuming and costly, making it less practical for smaller medical applications [23, 42]. Concerns about the ChatGPT training datasets have also been raised, causing possible biases. This effect could not only possibly limit its capabilities and could produce counterfactual results [43, 44]. 

Limitations

This review has limitations in the context of searching in multiple databases. Since ChatGPT is a relatively new topic, some preprints were included. Other papers that PubMed does not yet index could have been missed. Furthermore, we included articles published before November 2022 to reflect on AI chatbots, given that the available data on ChatGPT was still limited during our literature review. 

## Conclusions

In conclusion, this review offers a comprehensive summary of the early presence of ChatGPT in the medical literature during the first four months following its launch. It highlights the growing body of literature on the adaptations and healthcare responses to this transformative AI model. The publications on ChatGPT in medical indexing platforms provide a valuable overview of its potential applications in medical education, scientific research, medical writing, and diagnostic decision-making. However, they also raise ethical concerns, such as authorship, accountability, transparency, and the potential for bias or misinformation. To maintain the integrity of health science publications, specific criteria for including ChatGPT as a co-author should be established by publishers. Nevertheless, more research is needed to evaluate the effectiveness and ethical implications of using AI chatbots like ChatGPT across different disciplines.
